# Optimal antibiotics duration following surgical management of septic olecranon bursitis: a 12-year retrospective analysis

**DOI:** 10.5194/jbji-9-107-2024

**Published:** 2024-03-06

**Authors:** Said El Zein, Elie F. Berbari, Allison M. LeMahieu, Anil Jagtiani, Parham Sendi, Abinash Virk, Mark E. Morrey, Aaron J. Tande

**Affiliations:** 1 Division of Public Health, Infectious Diseases and Occupational Medicine, Department of Medicine, Mayo Clinic, Rochester, MN, USA; 2 Department of Quantitative Health Sciences, Mayo Clinic, Rochester, MN, USA; 3 Department of Infectious Diseases, Kaiser Permanente Southern California, Fontana, CA, USA; 4 Institute for Infectious Diseases, University of Bern, Bern, Switzerland; 5 Department of Orthopedic Surgery, Mayo Clinic, Rochester, MN, USA

## Abstract

**Introduction**: The absence of a standardized postoperative antibiotic treatment approach for patients with surgically treated septic bursitis results in disparate practices. **Methods**: We retrospectively reviewed charts of adult patients with surgically treated septic olecranon bursitis at Mayo Clinic sites between 1 January 2000 and 20 August 2022, focusing on their clinical presentation, diagnostics, management, postoperative antibiotic use, and outcomes. **Results**: A total of 91 surgically treated patients were identified during the study period. *Staphylococcus aureus* was the most common pathogen (64 %). Following surgery, 92 % (84 of 91 patients) received systemic antibiotics. Excluding initial presentations of bacteremia or osteomyelitis ( n=5 ), the median duration of postoperative antibiotics was 21 d (interquartile range, IQR: 14–29). Postoperative complications were observed in 23 % (21 of 91) of patients, while cure was achieved in 87 % (79 of 91). Active smokers had 4.53 times greater odds of clinical failure compared with nonsmokers (95 % confidence interval, 95 % CI: 1.04–20.50; p=0.026 ). The highest odds of clinical failure were noted in cases without postoperative antibiotic administration (odds ratio, OR: 7.4). Conversely, each additional day of antibiotic treatment, up to 21 d, was associated with a progressive decrease in the odds of clinical failure (OR: 1 at 21 d). **Conclusion**: The optimal duration of antibiotics postoperatively in this study was 21 d, which was associated with a 7.4-fold reduction in the odds clinical failure compared with cases without postoperative antibiotics. Further validation through a randomized controlled trial is needed.

## 1 Introduction

Septic olecranon bursitis is commonly encountered in clinical practice (Laupland and Davies, 2001). The superficial location of the olecranon bursa makes it susceptible to injuries from trauma and repetitive microtrauma, which can result in contamination by skin pathogens and subsequent infection (Reilly and Kamineni, 2016; Lormeau et al., 2019). Given its limited blood supply, hematogenous seeding of the bursa is uncommon (Blackwell et al., 2014; Reilly and Kamineni, 2016; García-Porrúa et al., 1999). Patients are often managed medically in the outpatient setting, although hospital admission and surgical management are sometimes required for complicated or refractory cases. Estimates suggest that both septic olecranon and prepatellar bursitis account for 0.01 % to 0.1 % of all hospital admissions (Mcafee and Smith, 1988), with half of these patients necessitating surgical intervention (Sayegh and Strauch, 2014). While surgical indications are clearly outlined in certain situations, there is still a notable void in the existing literature when it comes to postoperative care, particularly concerning the administration of antibiotics, which varies considerably among different healthcare institutions (Baumbach et al., 2013a, b, 2014; Ho and Su, 1981; Perez et al., 2010; García-Porrúa et al., 1999).

We conducted a comprehensive review of patients who underwent surgical treatment for bacterial septic olecranon bursitis at our institution over a 12-year period. This involved evaluating their clinical profiles and management strategies, primarily focusing on the impact of postoperative antibiotic use and the optimal duration of postoperative antibiotics on their clinical outcomes.

## 2 Methods

### 2.1 Patient selection

We conducted a retrospective chart review of all patients 18 years and older who were diagnosed with septic olecranon bursitis that was managed surgically with bursectomy at Mayo Clinic sites between 1 January 2000 and 30 August 2022. The search criteria and search results are outlined in the Supplement and Fig. S1, respectively.

The diagnosis of septic olecranon bursitis was established based on the presence of clinical symptoms and physical exam findings, including fluctuance, redness, warmth, pain, sinus tract, and purulent drainage, coupled with a positive culture from either preoperative aspiration or an intraoperative specimen. Patients with negative cultures were included only if consistent symptoms and physical examination findings were documented in the medical record, in conjunction with the presence of intraoperative purulence, the absence of an alternative diagnosis, and a determination of septic bursitis as noted by an Orthopedic surgeon or an infectious disease specialist. The study excluded patients who did not have bursal fluid or tissue specimens submitted for cultures and those diagnosed with fungal and nontuberculous mycobacterial infection.

### 2.2 Data collection

Information pertaining to the patient's demographics, comorbidities, clinical presentation, laboratory markers, radiographic imaging, operative reports, microbiology, antibiotic therapy, and clinical outcomes were collected. All patients in this cohort underwent bursectomy, debridement, and irrigation. None of the patients underwent debridement and irrigation without bursectomy. Study definitions are outlined in the Supplement.

### 2.3 Statistical analysis

Patient characteristics were summarized using the median (interquartile range, IQR) for continuous variables and the frequency (percentage) for categorical variables. Imaging and laboratory characteristics were summarized similarly. The Mann–Whitney U test was used for comparisons of continuous variables between two independent groups when the assumptions of normality were not met. Univariate logistic regression models assessed the associations between patient characteristics and clinical failure. A univariate logistic regression model, including a cubic spline term for the total duration of antibiotics, was used to plot the relationship between total duration of antibiotics and clinical failure.

Complete case analysis was used to handle any missing data. Two-tailed p values of 0.05 or less were considered statistically significant. Data management and statistical analysis were performed in SAS 9.4 (SAS Institute Inc, Cary, North Carolina).

## 3 Results

### 3.1 Patient characteristics

We included 91 patients who underwent bursectomy for septic olecranon bursitis. Their demographics are detailed in Table 1. Their median age was 62 years (IQR: 51–74), and the majority were men (68 %). A total of 31 patients had an autoimmune disorder (34 %), most commonly rheumatoid arthritis ( n=20 , 65 %). Moreover, 30 patients were immunocompromised owing to active malignancies (hematologic, n=3 ; solid tumors, n=4 ), hematopoietic stem cell transplantation ( n=1 ), solid-organ transplantation ( n=1 ), and pharmacologic immunosuppression ( n=26 ), most commonly with a regimen containing prednisone ( n=18 ), tumor necrosis factor- α (TNF- α ) inhibitor ( n=5 ), or methotrexate ( n=5 ).

**Table 1:** Patient characteristics.

*Characteristic*	Total ( N=91 )
Age, median (IQR)	62.0 (51.5–74.0)
Female sex – n (%)	29 (32 %)
Body mass index (BMI) (kg m^-2^), median (IQR)	28.2 (23.7–32.8)
Manual occupation – n (%)	8 (9 %)
Abrasions or wounds on the affected elbow – n (%)	16 (18 %)
History of surgery on the affected elbow – n (%)	16 (18 %)
Fall on the affected arm/trauma to elbow – n (%)	36 (40 %)
*Comorbidities* – n (%)	
Diabetes mellitus	17 (19 %)
End-stage renal disease	1 (1 %)
Autoimmune disease	31 (34 %)
Rheumatoid arthritis	20 (65 %)
Inflammatory bowel disease	5 (17 %)
Other	6 (19 %)
Hematologic malignancy	3 (3 %)
Solid malignancy	4 (4 %)
*Smoking history*, n (%)	
Never smoker	43 (47 %)
Current smoker	18 (20 %)
Former smoker	30 (33 %)
*Pharmacologic immunosuppression*, n (%)^*^	26 (29 %)
Prednisone	18 (69 %)
> 20 mg of prednisone equivalents for ≥ 2 weeks	4 (22 %)
TNF- α inhibitor	5 (19 %)
Methotrexate	5 (19 %)
Other	14 (54 %)
*Clinical presentation*	
Duration of symptoms prior to presentation, median (IQR), N=89	13.0 (4.0–53.0)
Fever (temperature > 38.3 °C) – n (%)	13 (14 %)
Local signs of inflammation at time of presentation – n (%)	
Mild–moderate swelling, redness, and/or pain	44 (48 %)
Moderate–severe swelling, redness, and/or pain	29 (32 %)
Extensive peribursal cellulitis	18 (20 %)
Presence of wound overlying the bursa – n (%)	
No open wound	31 (34 %)
Minor skin lacerations	5 (6 %)
Small open wound	23 (25 %)
Sinus tract/fistula	32 (35 %)
*Bursa imaging* – n (%)	17 (19 %)
Ultrasound showing fluid collection in the olecranon bursa	2 (12 %)
CT scan showing fluid densities in the subcutaneous tissues	4 (24 %)
CT scan showing hypodense bursa with wall enhancement	5 (29 %)
MRI showing bursal fluid collection with T1/T2 changes and gadolinium enhancement	9 (53 %)
MRI showing evidence of osteomyelitis	1 (6 %)
*Laboratory data within 48 h of surgery, median (IQR)*	
White blood cell count (WBC) ( $\times 10^{9}$ L^-1^), N=74	9.2 (7.2–11.9)
Erythrocyte sedimentation rate (ESR) (mm s^-1^), N=32	38.0 (16.0–60.5)
C-reactive protein (CRP) (mg L^-1^), N=42	58.3 (13.3–139.4)

^*^ Represents the number of times a specific immunosuppressive medication was used, whether alone or in combination with other medications, among the 26 patients who were receiving pharmacologic immunosuppression. Other medications include mycophenolate, azathioprine, tacrolimus, rituximab, tocilizumab, and chemotherapy

Overall, 60 patients (66 %) had mechanical factors predisposing them to septic bursitis, primarily due to elbow trauma ( n=36 , 40 %). Surgical intervention was mostly performed for a draining sinus or non-healing open wound ( n=37 , 40 %) followed by septic bursitis that was refractory to medical therapy ( n=35 , 39 %). Only 13 patients (14 %) had a fever within 48 h of surgery. The median duration of symptoms prior to surgery was 13 d (IQR: 4–53) with a longer duration of symptoms reflecting the presence of a draining sinus or persistent/recurrent episodes of septic bursitis failing conservative medical management.

### 3.2 Imaging and laboratory data

Elbow X-rays were standard for most patients. In 17 cases (18.7 %), additional radiographic imaging was obtained for additional infection assessment. In addition to the findings outlined in Table 1, two patients had signs of myositis on magnetic resonance imaging (MRI), while one patient had computed tomography (CT) findings raising concerns for necrotizing fascitis. Olecranon osteomyelitis was suspected on MRI in one patient and on an elbow X-ray in another patient. Notably, two patients had no preoperative imaging, but the suspicion of osteomyelitis arose during the surgery due to the presence of soft bone at the infected tissue base.

When tested, most patients had elevated inflammatory markers within 48 h preceding surgery. The median erythrocyte sedimentation rate (ESR) was 38 mm h^-1^ (IQR: 16–60.5; reference range: 0–29 mm h^-1^), and the median C-reactive protein (CRP) level was 58.3 mg L^-1^ (IQR: 13.3–139.4; reference range: ≤ 8.0 mg L^-1^). In contrast, most patients did not have elevated total white blood cell (WBC) counts, with a median of $9.2\times 10^{9}$ L^-1^ (IQR: 7.2–11.9; reference range: 3.4–9. $6\times 10^{9}$ L^-1^).

Table 2 details microbiological findings. All patients received preoperative antibiotics for surgical site infection prophylaxis, as per institutional protocol. Blood cultures were positive in 3 of 48 (6 %) patients at the time of surgical admission, all growing methicillin-susceptible *Staphylococcus aureus* (MSSA). Bursal fluid Gram stain was performed in 79 patients and was positive in only 29 cases (37 %). The most isolated organism was *S. aureus *(58 of 84 patients, 69 %), with the majority being MSSA (43 of 58, 74 %). Seven patients had polymicrobial infection, six of which had clinical evidence of septic bursitis associated with a fistula or open wound at the time of presentation. Among seven patients with negative bursal fluid cultures, three had no recent antibiotic use but presented with pain, redness, and swelling. Intraoperative findings of purulent fluid ( n=1 ), tissue that appeared infected ( n=1 ), and surgeon impression ( n=1 ) were consistent with septic bursitis.

**Table 2:** Microbiological data and clinical management.

*Characteristic*	Overall ( n=91 )
Positive blood cultures on admission, n (%), N=48	3 (6 %)
Positive bursal aspirate stain, n (%), N=79	29 (37 %)
Bursal aspirate stain result, n (%), N=29	
Gram-positive cocci	29 (100 %)
Bursal fluid cultures – n (%)	
*Staphylococcus* spp.	66 (73 %)
Methicillin-susceptible *S. aureus*	43 (47 %)
Methicillin-resistant *S. aureus*	15 (16 %)
Coagulase-negative staphylococci	8 (9 %)
*Streptococcus* spp.	7 (8 %)
Gram-negative bacilli^a^	2 (2 %)
Polymicrobial growth	7 (8 %)
Other	2 (2 %)
No growth	7 (8 %)
*Management* – n (%)	
Bursal fluid aspiration prior to surgery	36 (39.5 %)
Bursectomy performed	
One-stage procedure	89 (98 %)
Two-stage procedure	2 (2 %)
Indication for surgery	
Septic bursitis refractory to medical therapy	35 (39 %)
Abscess and/or extensive surrounding infection requiring debridement	18 (20 %)
Draining sinus or non-healing open wound	37 (40 %)
Foreign body	1 (1 %)
Required admission and inpatient management	57 (63 %)
Duration of hospitalization (days), median (IQR), N=62	5.0 (3.0–7.0)
IV antibiotics administered during the course of treatment^c^	60 (66 %)
Empiric IV antibiotics used on admission, n (%), $N=60^{b}$	
Vancomycin	44 (73 %)
Cefazolin	17 (28 %)
Ceftriaxone	14 (23 %)
Daptomycin	1 (2 %)
Ertapenem	0 (0 %)
Meropenem	2 (3 %)
Cefepime	7 (12 %)
Piperacillin/tazobactam	15 (25 %)
IV antibiotics tailored to bursal culture results, n (%), N=60	
Vancomycin	11 (18 %)
Cefazolin	10 (17 %)
Ceftriaxone	13 (22 %)
Daptomycin	4 (7 %)
Ertapenem	4 (7 %)
Cefepime	3 (5 %)
Piperacillin/tazobactam	0 (0 %)
Duration of IV antibiotics, N=60	18.5 (6.0–29.0)
Oral antibiotics administered as part of the definitive treatment course	57 (62.6 %)
Amoxicillin	3 (5 %)
Amoxicillin–clavulanate	4 (7 %)
Fluoroquinolone	10 (17 %)
Cefadroxil	9 (16 %)
Cephalexin	13 (23 %)
Trimethoprim–sulfamethoxazole	8 (14 %)
Linezolid	1 (2 %)
Doxycycline/minocycline	9 (16 %)
Duration of oral antibiotics, median (IQR), N=57	14.0 (10.0–21.0)
Total duration of antibiotic therapy, median (IQR)	21.0 (14.0–30.0)

^a^ *Pseudomonas aeruginosa* and *Klebsiella oxytoca*. ^b^ Does not represent the full regimen used, rather only the number of times a specific antibiotic was used (whether alone or in combination with other antibiotics). ^c^ Excluding patients who received only one dose of preoperative antibiotics for surgical site infection prophylaxis.

### 3.3 Clinical management

Following surgery, 84 of 91 patients (92 %) received systemic antibiotics. Excluding five patients with osteomyelitis or positive blood cultures on presentation, the median duration of postoperative antibiotics administered was 21 d (IQR: 14–29). Vancomycin, alone or combined with other antibiotics, was used in the majority of patients started on intravenous (IV) antibiotics on admission (44 of 60 cases, 73 %). Oral antibiotics were used for the full duration in 25 of 84 patients (30 %), while IV antibiotics were used for the full duration in 27 of 84 (32 %) patient. A transition from IV to oral antibiotics was implemented for 32 of 84 patients (38 %). Modifications to the antibiotic regimen were based on bursal culture, as detailed in Table 2. One immunocompromised patient with rheumatoid arthritis was placed on long-term suppression with doxycycline (3 months) due to a history of multifocal staphylococcal infections and poor wound healing postoperatively.

### 3.4 Operative complications and outcomes

Postoperative complications occurred in 21 of 91 (23 %) patients (Table 3). Among patients requiring flap coverage, four had a sinus tract on initial presentation and one had both a sinus tract and osteomyelitis. Preemptive flap coverage was performed in two patients, while the remaining were performed due to poor wound healing postoperatively. Excluding the patient with osteomyelitis on presentation, flap recipients received a longer course of antibiotics (median: 43 d; IQR: 29–50) compared with those who did not require a flap (median: 20 d; IQR: 13–28) ( p=0.004 ).

**Table 3:** Outcomes.

Duration of follow-up (days), median (IQR)	51.0 (25.5–93.0)
Postoperative complications, n (%)	21 (23 %)
Prolonged/persistent pain, n (%), N=91	5 (5 %)
Fistula formation, n (%), N=91	2 (2 %)
Delayed wound healing, n (%), N=91	12 (13 %)
Cellulitis at the site of incision, n (%), N=91	3 (3 %)
Flap coverage, n (%), N=91	6 (7 %)
Negative-pressure wound therapy, n (%), N=91	3 (3 %)
Clinical failure^*^, N=91	12 (13 %)
Recurrent or secondary bursal infection, n (%)	8 (67 %)
Osteomyelitis, n (%)	4 (33 %)

^*^ Composite failure defined as the recurrence of the infection with the same organism, reinfection with another organism, or a local complication, such as osteomyelitis, that was not present at the time of diagnosis within 90 d of surgery.

Treatment failure was documented in 12 of 91 patients (13 %), with a median follow-up of 131 d (IQR: 109.5–190.7). Failures were due to the recurrence of the infection with the same organism ( n=3 ), reinfection with a different organism ( n=5 ), and osteomyelitis that was not present or suspected on initial presentation ( n=4 ). Four of the patients who failed did not receive antibiotics following bursectomy, and failure occurred after a median of 35 d postoperatively. Among those treated with postoperative antibiotics, failures occurred a median of 44 d after the discontinuation of antibiotics. Only one patient with negative cultures on presentation failed treatment and had a recurrence of septic bursitis with methicillin-resistant *S. aureus* (MRSA) 3 months following the index procedure.

On univariate analysis, active smokers had 4.53 times greater odds of clinical failure compared with patients who never smoked (95 % confidence interval, 95 % CI: 1.04–20.50; p=0.026 ). Neither the presence of an underlying autoimmune disorder (OR: 2.92; 95 % CI: 0.84–10.15) nor the existence of an immunosuppressive condition (OR: 2.08; 95 % CI: 0.61–7.14) were associated with an increased risk of failure; however, this might be due to the study's insufficient power to detect a significant association (Table S1 in the Supplement).

**Figure 1:**
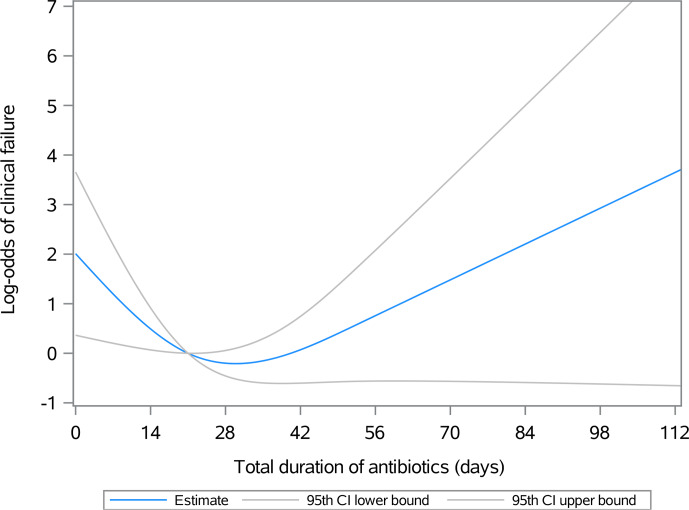
Association between the duration of postoperative antibiotic therapy and the natural logarithm of the odds ratio (log-odds, OR) for clinical failure in patients with olecranon septic bursitis treated surgically. By day 21, the log-odds of clinical failure approach zero and the lower and upper bounds of the 95th confidence interval converge. Beyond 21 d, the confidence interval for the log-odds estimates spans values both less than and greater than zero, indicating that the association is no longer statistically significant. The odds can be calculated as $=e^{\text{log odds}}$ , where e is the base of the natural logarithm.

Figure 1 illustrates the relationship between the duration of postoperative antibiotic therapy and the natural logarithm of the odds ratio (log-odds) for clinical failure in surgically treated patients with septic olecranon bursitis, excluding those with initial osteomyelitis or bloodstream infections. Not administering postoperative antibiotics (represented by the y intercept) correlates with the highest risk of clinical failure, indicated by a log-odds ratio of 2 (OR: 7.38). Each additional day of antibiotic treatment reduces the odds of clinical failure; at 21 d, the log-odds of clinical failure approach zero (OR: ≈ 1). However, extending antibiotic administration beyond 21 d does not result in a statistically significant further reduction in the odds of clinical failure. Due to the small sample size and study design, stratification of the outcomes based on the route of antibiotics was not possible.

## 4 Discussion

In this study, 79 patients (87 %) achieved clinical cure after undergoing surgery for septic olecranon bursitis, and 21 patients (23 %) experienced noninfectious postoperative surgical complications. On univariate analysis, only a history of active smoking was associated with increased odds of clinical failure. Factors such as existing comorbidities, immunosuppression, and initial infection severity did not significantly influence clinical failure rates. Notably, our results suggests that postoperative antibiotic administration up to 21 d was associated with a statistically significant incremental reduction in the odds of clinical failure.

Our findings align with previous reports on septic olecranon bursitis. Specifically, most of the patients in this study were men (68 %) who had an identified risk factor for olecranon bursitis due to trauma, recurrent microtrauma, or surgery to the affected elbow (Laupland and Davies, 2001; Reilly and Kamineni, 2016). Moreover, compared with patients who never smoked, active but not former smokers were at increased odds of clinical failure following bursectomy. This association may be partly due to the bursa's watershed midline blood supply (Blackwell et al., 2014), which, when combined with the temporary effects of smoking that decrease tissue oxygenation and disrupt the inflammatory healing response, leads to delayed wound healing and an elevated risk of postoperative infectious complications (Sørensen, 2012; Skedros et al., 2023). Notably, smoking cessation for 4 weeks or more appears to partially reverse these effects (Sørensen, 2012), highlighting the importance of patient counseling and the implementation of effective strategies to facilitate and maintain smoking cessation in the perioperative period.

There is no established clinical or laboratory finding that is specific for diagnosing septic olecranon bursitis. While leukocytosis, elevated ESR, and CRP levels are seen in septic cases, these markers are nonspecific and can also be elevated due to trauma, inflammation, or noninfectious conditions such as crystal deposition disease (Reilly and Kamineni, 2016; Lormeau et al., 2019; García-Porrúa et al., 1999). Although 84 of 91 (92 %) of our patients had culture-confirmed septic bursitis, only 13 of 91 (16 %) had a fever and most did not have WBC elevation, underscoring the limited diagnostic value of these markers for septic bursitis.

Consistent with published literature (Reilly and Kamineni, 2016; Lormeau et al., 2019; Uçkay et al., 2017), *S. aureus* was the predominant causative organism for septic olecranon bursitis in this study ( n=58 , 69 %). Along with coagulase-negative staphylococci and streptococci, these organisms accounted for the majority ( n=73 , 87 %) of the culture-positive cases. In a 2010 study from Switzerland, only 3 of 217 (1.4 %) of the *S. aureus *isolates responsible for olecranon and patellar bursitis were MRSA (Perez et al., 2010). This contrasts with 15 of 58 (26 %) of the patients in our cohort. Moreover, although less common, polymicrobial infections and infections caused by Gram-negative bacilli were identified in our cohort, particularly in patients with open wounds or draining sinus tracts, highlighting the importance of obtaining bursal cultures in surgical cases. Therefore, while empirical treatment should primarily target MSSA and streptococci, consideration for Gram-negative and/or MRSA coverage should be based on patient-specific factors, such as presence of open wounds, and tailored with final culture results.

The optimal duration of postoperative antibiotics remains uncertain. In a study by Baumbach et al. (2013a) surveying Swiss infectious diseases and orthopedic surgeons regarding the management of septic bursitis, a range of postoperative antibiotic durations were reported, varying between 3 and 28 d. Moreover, 25 of the 92 (27 %) surveyed physicians did not routinely prescribe antibiotics postoperatively (Baumbach et al., 2013a). In our cohort, excluding initial bacteremia or osteomyelitis, the median postoperative antibiotic course was 21 d, although with wide variability in both the route and duration of antibiotic treatment, ranging from 0 to 102 d. In a study by Perez et al. (2010), a short antibiotic course ( ≤ 7 d) following bursectomy for olecranon or prepatellar septic bursitis in immunocompetent patients was not associated with an increased risk of infection relapse compared with longer courses (8–14 or > 14 d). In a randomized-controlled evaluation of one- vs. two-stage bursectomy for septic olecranon and prepatellar bursitis, one-stage bursectomy was associated with a similar risk of treatment failure to two-stage bursectomy when a 7 d course of postoperative antibiotics was administered (10 % vs. 16 %). This trial, however, was not designed to assess the impact of postoperative antibiotic durations on failure rates (Uçkay et al., 2017). Moreover, all patients in both studies received postoperative antibiotics; therefore, the effect of not administering postoperative antibiotics on the risk of failure could not be assessed. Our study, excluding patients with osteomyelitis and bloodstream infection on presentation, identified increased odds of clinical failure when no postoperative antibiotics were administered. Moreover, in contrast to prior studies, each additional day of antibiotics up to 21 d, was associated with a further decreased risk of failure. An explanation for these discrepant results potentially lies in the study populations and methodologies, possibly limiting comparability. For example, we predominantly included patients with culture-positive septic olecranon bursitis ( n=84 , 92 %), whereas prior studies included a larger number of culture-negative cases (up to 25 %). Moreover, our study was strictly limited to cases of olecranon bursitis, excluding prepatellar cases. Although increasing evidence indicates that oral antibiotic therapy is not inferior to IV therapy for treating complex orthopedic infections (Li et al., 2019), our study's small sample size and design limitations prevented us from making definitive conclusions about clinical outcomes based on the route of antibiotic administration.

Despite the variability in treatment practices between institutions, the surgical indications for septic olecranon bursitis have not significantly changed over the last 2 decades. Surgery is typically reserved for select cases such as those involving refractory or relapsing infection, critically ill patients, presence of extensive peribursal soft tissue involvement requiring debridement, presence of a bursal abscess that fails needle aspiration, or for patients with chronic draining sinus (Baumbach et al., 2014; Small and Ross, 2005; Zimmermann et al., 1995). The ideal surgical approach (incision and drainage vs. bursectomy) remains debated (Lormeau et al., 2019; Baumbach et al., 2013a). In patients undergoing bursectomy, a one-stage procedure was effective, safe, and associated with lower rates of wound dehiscence compared with the two-stage approach (Uçkay et al., 2017). In our study, most patients underwent one-stage bursectomy ( n=89 , 98 %) and none underwent incision and drainage alone. While we could not compare the outcomes between various surgical procedures, clinical cure was achieved in 79 of 91 (87 %) cases, while postoperative surgical complications were noted in only 21 of 91 (23 %) cases.

This study has inherent limitations. First, our cohort is sourced from a quaternary referral center, possibly not fully reflecting septic olecranon bursitis management and outcomes nationwide. Second, although most patients had baseline standard radiographs, initial osteomyelitis diagnoses may have been overlooked and potentially misinterpreted as treatment failures following postoperative osteomyelitis identification. Third, reliance on International Classification of Diseases (ICD) and Current Procedural Terminology (CPT) codes for patient identification introduces potential inaccuracies. Furthermore, while we created a more uniform group by applying strict criteria for the inclusion of culture-negative cases and excluding patients without culture data, we may have inadvertently omitted some cases of septic bursitis. Fourth, our study was limited to cases that were managed surgically; therefore, it was not designed to compare clinical characteristics and outcomes between conservatively managed patients and those undergoing surgery. Such a comparison might have provided additional insights. Finally, self-reported variables such as smoking and drug use history are susceptible to reporting bias. Moreover, retrospective data collection is subject to the inherent limitations of medical record accuracy. The team made efforts to ensure data precision and reduce the risk of information bias.

## 5 Conclusion

Our findings suggest that, in patients with septic olecranon bursitis managed surgically, the administration of postoperative antibiotics is associated with reduced odds of clinical failure, and a prolonged course of antibiotics, potentially up to 21 d, may be beneficial. While not all patients will require an extended antibiotic regimen post-surgery, our results underscore the need for a randomized controlled trial to substantiate these findings and aid in identifying patients who could benefit most from either a shorter or longer treatment regimen. Furthermore, perioperative counseling and strategies to support smoking cessation should be offered to all active smokers, given that smoking is associated with poor wound healing and was found to increase the odds of clinical failure following bursectomy in our study.

## Supplement

10.5194/jbji-9-107-2024-supplement The supplement related to this article is available online at: https://doi.org/10.5194/jbji-9-107-2024-supplement.

## Data Availability

The dataset with all identifying information removed is available from the corresponding author upon reasonable request.

## Code availability

The codes used to perform the statistical analysis can be shared by the corresponding author upon reasonable request.
