# Supplementary material for: Optimal antibiotics duration following surgical management of septic olecranon bursitis: a 12-year retrospective analysis
Source: J Bone Jt Infect. 2024 Mar 6;9(2):107–15. doi: 10.5194/jbji-9-107-2024 (PMC11110802; doi:10.5194/jbji-9-107-2024)
Supplement: The supplement related to this article is available online at: https://doi.org/10.5194/jbji-9-107-2024-supplement. [file jbji-9-107-supplement.pdf]

Supplement of J. Bone Joint Infect., 9, 107–115, 2024
https://doi.org/10.5194/jbji-9-107-2024-supplement
© Author(s) 2024. CC BY 4.0 License.

Supplement of

Optimal antibiotics duration following surgical management of septic olecranon bursitis: a 12-year retrospective analysis

Said El Zein et al.

Correspondence to: Aaron J. Tande (tande.aaron@mayo.edu)

The copyright of individual parts of the supplement might differ from the article licence.

Methods

Search criteria:

In order to generate an initial list of patients, we employed a combination of International Classification of Diseases (ICD) codes pertinent to bursitis (ICD-9 726.33 and ICD-10 M70.21, M70.22) and the Current Procedural Terminology (CPT) code specific to olecranon bursa excision (CPT 24105). To detect surgically treated patients who might not have been identified using the CPT code alone, we separately conducted a document text search within the medical records of patients diagnosed with bursitis, utilizing keywords such as "bursectomy" and "debridement".

Definitions

Immunosuppression was defined as the presence of one or more of the following comorbidities: active solid or hematologic malignancy, HIV infection with CD4 count < 200 / mm3 , history of receiving a solid organ or hematopoietic stem cell transplant, primary immunodeficiency, or the administration of pharmacologic immunosuppressive therapy due to an underlying malignancy or autoimmune disorder.

Smoking history was determined from medical records, based on patient self-reporting. Our institution routinely assesses smoking history during each hospital admission. Current or active smoking was defined as self-reported smoking within one month prior to hospital admission for the index surgical procedure. Former smoking was defined as a history of smoking but not within the one-month preceding hospital admission for the index surgical procedure. Clinical cure and failure were assessed at the time of last follow-up by an Infectious Disease provider and/or Orthopedic Surgeon with a documented physical examination and clinical status on record. Clinical cure was defined as complete resolution of signs and symptoms of septic bursitis without further need for surgical intervention or antibiotic therapy after completion of the initial treatment course following the index surgical procedure. Clinical failure was defined as reinfection with the same or different pathogen and requiring an additional course of antibiotics with or without the need for additional tissue debridement, or new diagnosis of osteomyelitis that was not evident or suspected, either radiographically or intraoperatively, at the time of surgery.

Non-infectious post-operative complications such as delayed wound healing, fistula formation without concurrent infection that required additional antibiotic therapy, simple and limited superficial surgical site infection, and the need for flap coverage (whether preemptive or due to poor wound healing without concurrent infection) were not classified as treatment failures.

Supplementary table

Table S1: Unadjusted logistic regression, outcome – clinical failure

Characteristic	OR	95% CI	p-value
Age	1.00	(0.81, 1.22)	0.981
Sex	0.96	(0.26, 3.50)	0.951
Race	-	-	-
BMI	0.98	(0.88, 1.09)	0.758
Smoking history			0.081
Active smoker	4.63	(1.04, 20.50)	**0.026**
Former smoker	1.03	(0.21, 4.98)	0.303
Comorbidities			
Diabetes	0.39	(0.05, 3.28)	0.385

ESRD requiring HD or PD	-	-	-
Liver disease or cirrhosis	-	-	-
Injectable drug use	-	-	-
Autoimmune disorder	2.92	(0.84, 10.15)	0.092
Immunosuppressive condition	2.08	(0.61, 7.14)	0.243
Duration of symptoms prior to admission	0.97	(0.91, 1.04)	0.343
Local signs of inflammation on presentation			0.838
Moderate-severe swelling, redness and/or pain	1.29	(0.31, 5.29)	0.974
Extensive local infection with peribursal cellulitis	1.59	(0.33, 7.53)	0.647
WBC 48 hours before bursectomy	0.96	(0.81, 1.13)	0.599
ESR within 48 hours of bursectomy	0.99	(0.83, 1.19)	0.935
CRP within 48 hours of bursectomy	0.96	(0.90, 1.03)	0.286
Imaging performed	0.39	(0.05, 3.27)	0.385

OR for age is per 5 years. OR for duration of symptoms is per 5 days. OR for ESR is per 5 mm/hs. CRP is per 5 mg/L.

Figure S1: Patient identification

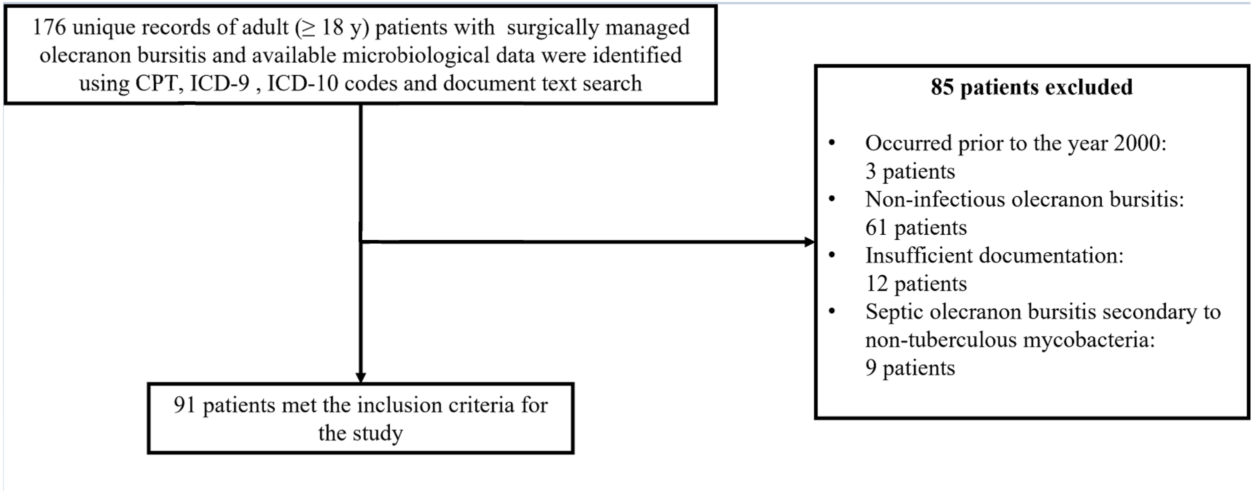

Patients with negative bursal fluid cultures, who had an alternative cause for their bursitis such as gout, pseudogout, or inflammatory arthritis related to an autoimmune disease, and/or those deemed not to have septic bursitis by the Orthopedic Surgeon and/or Infectious Disease physicians based on a combination of intraoperative and clinical findings, were diagnosed with aseptic (non-infectious) olecranon bursitis and were excluded from the analysis.
